# Fractions from Sea Buckthorn Seeds and Their Bioactive Ingredients as Modulators of Human Blood Platelet Response In Vitro: The Role of Thermal Processing

**DOI:** 10.3390/nu17193074

**Published:** 2025-09-27

**Authors:** Natalia Sławińska, Luiza Janko, Jerzy Żuchowski, Beata Olas

**Affiliations:** 1 Department of General Biochemistry, Faculty of Biology and Environmental Protection, University of Lodz, 90-236 Łódź, Poland; natalia.slawinska@edu.uni.lodz.pl (N.S.); luiza.janko@edu.uni.lodz.pl (L.J.); 2 Department of Biochemistry and Crop Quality, Institute of Soil Science and Plant Cultivation—State Research Institute, Czartoryskich 8, 24-100 Puławy, Poland; jzuchowski@iung.pulawy.pl

**Keywords:** blood platelet activation, hemostasis, sea buckthorn seeds, thermal processing

## Abstract

**Background:** Given the pivotal role of diet in cardiovascular diseases (CVDs), there is a growing demand for new sources of bioactive phytochemicals that can contribute to CVD prevention and treatment. Previous research has unveiled the cardioprotective properties of several parts of sea buckthorn (*Hippophae rhamnoides* L.). For example, various fractions isolated from raw and roasted sea buckthorn seeds showed antioxidant properties in vitro. In addition, the serotonin-rich fraction obtained from roasted seed extract had the strongest antioxidant activity. However, it was unclear which chemical constituents contribute to the anti-platelet potential of sea buckthorn seeds. **Methods:** The anti-platelet activity of two fractions (fraction b and fraction c) from raw sea buckthorn seed extract, two fractions (fraction d and fraction g) from roasted sea buckthorn seed extract, and two chemical compounds—isorhamnetin 3-*O*-β-glucoside-7-*O*-α-rhamnoside (a major component of fraction b), and serotonin (5-HT, 5-hydroxytryptamine), present in fraction c was estimated in several in vitro assays. **Results:** Isorhamnetin 3-*O*-β-glucoside-7-*O*-α-rhamnoside significantly inhibited platelet activation. It lowered the exposition of the active form of GPIIb/IIIa on the surface of 20 μM ADP-stimulated platelets by about 26%. It also inhibited the exposition of P-selectin on the surface of 10 and 20 μM ADP-stimulated platelets. In addition, isorhamnetin 3-*O*-β-glucoside-7-*O*-α-rhamnoside (at 50 µg/mL) significantly prolonged the time of thrombus formation. The results also indicate that fractions d and g (from roasted seeds) are more effective anti-adhesive factors than fractions from raw sea buckthorn seeds. **Conclusions:** It can be suggested that sea buckthorn seeds can serve as a new source of anti-platelet compounds (especially derivatives of isorhamnetin) beneficial in CVD prevention and treatment; however, in vivo research is needed to clarify their mechanism of action, physiologically relevant concentrations, and therapeutic potential.

## 1. Introduction

Given the pivotal role of diet in cardiovascular diseases (CVDs), there is a growing need for new dietary sources of bioactive phytochemicals that can contribute to CVD prevention and enhance the quality of life [1,2]. The consumption of a diet rich in fruits and vegetables has been associated with a decreased risk of thrombus formation and other benefits for the cardiovascular system. Their beneficial properties are often associated with their content of phenolic compounds, which can exert anti-platelet activity by inhibiting blood platelet activation at different stages, including degranulation or adhesion [3,4,5,6].

Seeds are valuable components of a balanced diet. They are commonly added to bread (which is a staple food in many cuisines) and other bakery products (such as bread rolls, muesli bars, biscuits, or granola). The addition of seeds to these products can improve their nutritional content and increase the daily intake of various bioactive phytochemicals; in this context, seeds rich in nutrients and active compounds can serve as functional food [7,8,9]. However, high temperatures employed during food processing can alter the secondary metabolites and biological properties of seeds [10,11,12]. They can damage bioactive compounds, which may lead to decreased biological activity. On the other hand, high temperatures can also release active components from the food matrix, induce the formation of new compounds with different properties, or enhance bioavailability [12,13,14].

**Figure 1:**
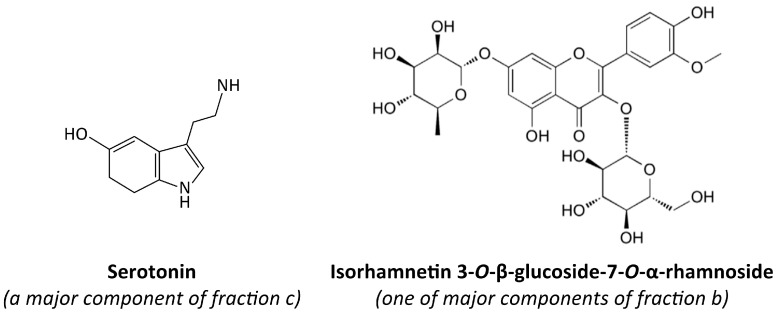
The chemical structures of serotonin and isorhamnetin 3-*O*-β-glucoside-7-*O*-α-rhamnoside [26,27].

Recent research has unveiled the cardioprotective properties of various organs of sea buckthorn (*Hippophae rhamnoides* L.), which are associated with its bioactive compounds, especially phenolics [15,16,17,18,19,20,21,22]. However, the most-often studied sea buckthorn parts are whole fruits and oil, while there is less information about the seeds. In particular, the effect of preparations from sea buckthorn seeds on hemostasis, and the mechanism of their anti-platelet activity remains poorly understood. Previous in vitro results showed that not only the extract from raw, but also the extract from roasted sea buckthorn seeds has anti-platelet, anticoagulant, and antioxidant potential [23,24]. Fractions isolated from these extracts (extracted with 1.5, 20, 66, or 85% methanol) also showed antioxidant properties in vitro [25]. However, it was unclear which chemical constituents contribute to the anti-platelet potential of sea buckthorn seeds. Therefore, the aim of this study was to estimate the in vitro anti-platelet activity of two fractions from raw sea buckthorn seed extract (fraction b and fraction c), and two fractions from roasted sea buckthorn seed extract (fraction d and fraction g). Each fraction contained a different set of chemical compounds. Total Thrombus formation Analysis System (T-TAS) PL-chip was used to evaluate the impact of the fractions on platelet thrombus formation. Flow cytometry was employed to monitor platelet activation by assessing the exposition of P-selectin and the active form of GPIIb/IIIa on the surface of platelets stimulated with ADP. The anti-adhesive activity of the fractions and their ability to inhibit cyclooxygenase-1 (COX-1) were assessed as well. The anti-platelet potential of two chemical compounds—isorhamnetin 3-*O*-β-glucoside-7-*O*-α-rhamnoside, which is one of major components of fraction b, and serotonin (5-hydroxytryptamine, 5-HT), present in large quantities in fraction c [25] was also evaluated. Aspirin was used as a reference anti-platelet compound. The chemical structures of serotonin and isorhamnetin 3-*O*-β-glucoside-7-*O*-α-rhamnoside are depicted in Figure 1.

## 2. Materials and Methods

### 2.1. Chemicals

PL-chips and other equipment needed for the T-TAS assay were acquired from Bionicum Sp. z o.o. (Warsaw, Poland). Serotonin was purchased from Thermo Fisher Scientific (Waltham, MA, USA). Methanol (isocratic grade), aspirin, tris(hydroxymethyl)aminomethane (Tris), phosphate-buffered saline (PBS), 4-nitrophenyl phosphate, bovine serum albumin (BSA), adenosine diphosphate (ADP), thrombin, collagen, and fibrinogen were purchased from Merck (Darmstadt, Germany). Antibodies (CD62P/PE, CD61/PerCP, and PAC-1/FITC) were from Becton Dickinson (Franklin Lakes, NJ, USA). The kit for measuring COX-1 activity was acquired from Cayman Chemical (Ann Arbor, MI, USA). All the other reagents were acquired from commercial suppliers, for example, POCH (Avantor performance materials, Gliwice, Poland) and Chempur (Piekary Śląskie, Poland). Isorhamnetin 3-*O*-β-glucoside-7-*O*-α-rhamnoside was purified according to the method described by Żuchowski et al. [27].

### 2.2. Plant Material

Sea buckthorn fruits were acquired from a horticultural farm in Sokółka, Podlaskie Voivodship, Poland (53° 24′ N, 23° 30′ E). The identification of the plant material was carried out by Mr. Stanisław Trzonkowski, the plantation owner. Whole branches with ripe fruits were harvested in the second half of August 2017. A voucher specimen (IUNG/HRH/2017/1) was deposited at the Department of Biochemistry and Crop Quality, Institute of Soil Science and Plant Cultivation, State Research Institute, Puławy, Poland. The fruits were picked manually and frozen. The seeds were isolated by homogenizing the fruits in water (with a kitchen blender), washing away skins and pulp with tap water, and leaving the obtained material to sediment. After the sedimentation, seeds were rinsed with methanol to facilitate drying, air-dried, and stored at room temperature.

A portion of the seeds was thermally processed in a bakery company, as described in a previous work [23]. The applied roasting method was chosen to imitate the conditions used in the baking of rye bread. First, the seeds were roasted in the baking chamber at 280 °C for approximately 10 min at 35% relative humidity; then, the temperature was lowered to 175 °C. The total roasting duration was 36 min.

### 2.3. Preparation of Fractions b, c, d, and g, and Their Chemical Characteristics

Fractions were prepared from the extracts from raw and roasted sea buckthorn seeds; isolation of the extracts was described in detail in Sławińska et al. [23]. To obtain the fractions, the extracts were separated by reversed-phase liquid chromatography; the complex fractionation procedure is extensively described in the article of Sławińska et al. [25]. A total of 7 fractions were obtained—3 fractions from raw seeds (fraction a, b, and c) and 4 fractions from roasted seeds (fraction d, e, f, and g). Four fractions, each representing a different type of chemical compounds were chosen for hemostasis studies—fractions b, c, d, and g. Fraction b and fraction c were prepared from raw seed extract, and contained compounds eluted with 66%, and 20% methanol, respectively. Factions d and g were obtained from roasted sea buckthorn seed extract and contained compounds eluted with 85%, and 1.5% methanol, respectively. The fractions’ composition was analyzed by UHPLC-MS, with a UltiMate 3000RS (Thermo Fischer Scientific, Waltham, MA, USA) chromatographic system fitted with a charged aerosol detector (CAD) and an Impact II Q-TOF mass spectrometer (Bruker Daltonics, Bremen, Germany). Fraction b contained diverse flavonol glycosides, mostly 3-O-dihexoside-7-O-deoxyhexosides of kaempferol, quercetin, and isorhamnetin. Isorhamnetin 3-O-glucoside-7-O-rhamnoside was another major flavonoid constituent of fraction b; its peak constituted about 4.3% of the total peak area (CAD). Serotonin was the dominant compound of fraction c; The peak of serotonin (also containing a few co-eluting compounds, apparently present in much smaller amounts) constituted about 28.6% of the total peak area (CAD) of the fraction. Fraction c also contained different B-type proanthocyanidins, phenylalanine, tryptophan, methylgallic acid, and small amounts of uridine, guanosine, xanthosine (or their isomers). Fraction d consisted mainly of triterpenoid saponins, it contained also triterpenoids, and small amounts of lysophospholipids, putative hydroxylated fatty acids (oxylipins), glycerophospho-N-acylethanolamines (traces), and acylated flavonoids. Fraction g consisted of polar compounds, mainly dihexoses. It also contained a small amount of trihexoses, a dihexose derivative, and serotonin (the peak of the dihexose derivative and serotonin constituted less than 4.3% of the total peak area (CAD) of the fraction). A detailed description and discussion of the results of analysis of the tested fractions can be found in the article of Sławińska et al. [25].

### 2.4. Preparation of Stock Solutions of the Fractions and Tested Chemical Compounds for Bioassays

The fractions, serotonin and aspirin were dissolved in 5% DMSO, while isorhamnetin 3-*O*-β-glucoside-7-*O*-α-rhamnoside in 75% DMSO. The final concentration of DMSO in the samples did not exceed 0.05% (*v*/*v*) (for the fractions) and 0.75% (*v*/*v*) (for isorhamnetin 3-*O*-β-glucoside-7-*O*-α-rhamnoside); such low concentrations of DMSO do not affect its hemostatic properties (data not presented). The final concentrations of the fractions and compounds in the samples were 1 and 50 μg/mL.

### 2.5. Blood Samples and Isolation of Blood Platelets

Human whole blood was drawn from healthy volunteers at “Diagnostyka” blood collection center located at Brzechwy 7A Street, Lodz, Poland. The donors did not drink alcohol, smoke, or take any medication or supplements for two weeks prior to blood drawing. Blood was collected into tubes with benzyl-sulfonyl-D-Arg-Pro-4-amidinobenzylamide (BAPA) (for T-TAS) or with citrate/phosphate/dextrose/adenine (CPDA) anticoagulant (for the rest of bioassays) and immediately transported to the laboratory for testing.

All blood sample analyses were carried out in accordance with the guidelines of the Helsinki Declaration for Human Research. All participants signed an informed consent form one day before blood collection. All procedures were carried out with the consent of the Bioethics Committee at the University of Łódź (approval code: 2/KBBN-UŁ/III/2014).

The isolation of blood platelets from whole blood was carried out using differential centrifugation. Whole blood collected into CDPA tubes was centrifuged at 235× *g* for 12 min. Afterward, platelet-rich plasma was centrifuged at 1020× *g* for 15 min. The supernatant was discarded, and the remaining pellet was gently reconstituted in Barber’s buffer (0.014 M Tris, 0.14 M NaCl, 5 mM glucose, pH 7.4; a modification of Tyrode’s buffer). The number of platelets was determined by a spectrophotometric measurement (Spectrophotometer UV/Vis Helios alpha; Unicam, Cambridge, UK) at 800 nm and adjusted to 2.0 × 10^8^/mL [28,29].

### 2.6. Total Thrombus-Formation Analysis System (T-TAS; PL-Chip)

The T-TAS system (ZACROS Corporation, Bunkyo City, Japan) monitors the in vitro thrombus formation process in full blood. The PL-chip specifically assesses primary hemostasis, as the BAPA anticoagulant used in this assay inhibits secondary hemostasis by blocking thrombin and factor Xa activity. The PL-chip’s standardized blood flow chamber system contains 26 microcapillaries coated with type I collagen. The assay is conducted under a shear stress rate of 1500/s. These conditions cause platelet activation and the formation of platelet thrombi, which block the flow path. This process is monitored through measurements of pressure inside the flow path. Further information about this assay can be found in a paper of Hosokawa et al. [30].

First, blood was incubated with the fractions and extracts at 37 °C for 30 min. The control sample was incubated with 0.9% NaCl. Then, 330 μL of the samples were moved to the PL-chip’s reservoir. The pressure was recorded for 10 min, or until the Occlusion Time (the pressure of 60 kPa) was reached. The results were recorded as AUC_10_ (area under the curve recorded for 10 min). The AUC_10_ parameter monitors the parameters of thrombus formation—its growth, stability, and intensity.

### 2.7. Flow Cytometry (Measurement of Blood Platelet Activation in Whole Blood)

The effect of sea buckthorn seed fractions and compounds on blood platelet activation was measured with flow cytometry. Platelet activation was evaluated by measuring the exposition of P-selectin (CD62P) and the active form of GPIIb/IIIa (PAC-1) on blood platelet surface. First, fractions and compounds were pre-incubated with full blood at 37 °C for 15 min. Afterward, 10 or 20 μM ADP was added to the samples, and the incubation continued for another 15 min. Next, the samples were diluted 10-fold with PBS. 10 μL of each sample was transferred into flow cytometry tubes and incubated for 30 min at room temperature in the dark with 3 μL of each of the following antibodies: CD61/PerCP (cat. no. 347408), PAC-1/FITC (cat. no. 340507), and CD62P/PE (cat. no. 348107). Afterward, the samples were incubated with 1% CellFix at 37 °C for 1 h and analyzed with a LSR II Flow Cytometer (Becton Dickinson, San Diego, CA, USA). At least 5000 CD61/PerCP-positive objects were recorded. Blood platelets were identified based on positive CD61/PerCP antibody staining. For each sample, the percentages of PAC-1/FITC-positive and CD62/PE-positive platelets were calculated [31,32].

### 2.8. Blood Platelet Adhesion (Using Washed Blood Platelets)

The adhesion of thrombin-activated blood platelets to collagen and fibrinogen was based on the assay described by Bellavite et al. [33]; it is a static method that utilizes the activity of acid phosphatase. First, 96-well plates were coated with 100 μL of either 0.04 μg/mL collagen or 100 μg/mL fibrinogen for 24 h at 4 °C on a rocking shaker. After 24 h each well washed three times with TBS (pH 7.5); then 200 μL of 1% BSA (*w*/*v*) was added, and the plates were incubated at 37 °C for 2 h. The fractions and compounds were incubated with washed blood platelets for 30 min at 37 °C. After removing BSA from the 96-well plates and washing three times with TBS (pH 7.5) containing 0.1 mM MgCl_2_ and 0.1 mM CaCl_2_, 100 μL of the samples was added to the wells in triplicate. 50 μL of a mixture containing 0.6 U/mL thrombin, 3 mM CaCl_2_, and 3 mM MgCl_2_ in TBS was added to positive control and the samples. The final concentration of thrombin in the samples was 0.2 U/mL. 50 μL of TBS was added to the negative control. The samples were incubated at 37 °C for 1 h and washed with PBS three times. 150 μL of 0.1 M citrate buffer (pH 5.4) with 0.1% Triton X-100 (*v*/*v*) and 5 mM p-nitrophenyl phosphate was added to all wells, and the samples were incubated for 1 h at room temperature. 100 μL of 2 M NaOH was added to each well and absorbance was read at 405 nm with a SPECTRO-star Nano Microplate Reader (BMG LABTECH, Ortenberg, Germany). The results were presented as a % of positive control.

### 2.9. COX-1 Activity

COX-1 (human) Inhibitor Screening Assay Kit (Item No. 701070, Cayman Chemical) was used to assess the effect of the fractions and compounds on cyclooxygenase 1 (COX-1). Before starting the reaction, the Reaction Buffer and test tubes were pre-heated to 37 °C. A tube with COX-1 was placed in boiling water for three min to obtain the background value. 10 μL of inactive COX-1 was added to a tube with 160 μL of Reaction Buffer, 10 μL of Heme, and 10 μL of inhibitor vehicle (0.05% DMSO). 160 μL of Reaction Buffer, 10 μL of Heme, 10 μL of COX-1, and 10 μL of inhibitor vehicle were added to two tubes for the 100% Initial Activity value. 160 μL of Reaction Buffer, 10 μL of COX-1, 10 μL of Heme, and 10 μL of the fractions and compounds (at the final concentrations of 1 and 50 μg/mL) were mixed. The incubation of the samples was carried out at 37 °C for 30 min. Afterward, 10 μL of Arachidonic Acid was added to the tubes to start the reaction. The samples were incubated at 37 °C for exactly 2 min. To stop the reaction, a saturated Stannous Chloride solution was added to the samples and incubated at 37 °C for 15 min. Afterward, Background samples were diluted 100× with ELISA Buffer, while the 100% Initial Activity and COX-1 Inhibitor samples were diluted 2000× and 4000×. The standards (prepared according to the kit manual) and the samples were added to the ELISA plate at two dilutions; each dilution was added in duplicate. The blank, non-specific binding, maximum binding, and total activity samples were prepared as well. All necessary reagents were added according to the manufacturer’s instructions. The plate was incubated at 4 °C for 18 h. After the incubation, the wells were washed five times and 200 μL of Ellman’s reagent was added. Additionally, 5 μL of Tracer was added to Total Activity well. Development of the plate took place on an orbital shaker at room temperature in the dark. The plate was developed until the absorbance of Maximum Binding wells exceeded 0.3 (blank subtracted). The absorbance readings were taken at 412 nm using a SPECTROstar Nano Microplate Reader (BMG LABTECH, Germany). COX-1 activity was calculated according to the manufacturer’s manual.

### 2.10. Data Analysis

Statistical analyses were performed using Statistica 10 (StatSoft 13.3, TIBCO Software Inc., Palo Alto, CA, USA). Shapiro–Wilk test was used to check the data distribution, while Levene’s test was used to determine if the variance was homogenous. When the data exhibited normal distribution and homogenous variance, the differences between groups were assessed with one-way ANOVA with Tukey’s post hoc. In other cases, the Kruskal–Wallis test was applied. The power of the tests was assessed with Statistica 10. The results are presented as means ± SD or medians and interquartile ranges. The differences between groups were considered to be significant when *p* < 0.05. Dixon’s Q-test was applied to remove the outliers.

## 3. Results

### 3.1. Measurement of the Exposition of P-Selectin and the Active Form of GPIIb/IIIa on the Surface of Platelets (In Whole Blood)

**Figure 2:**
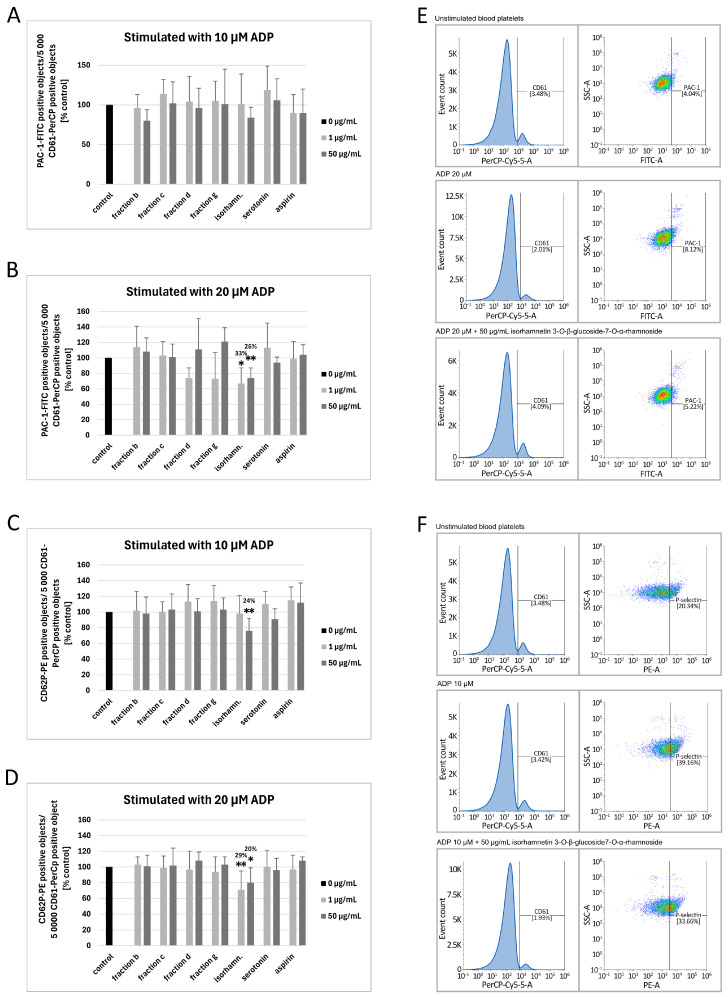
The effect of fraction b and fraction c from raw sea buckthorn seeds, fraction d and fraction g from roasted sea buckthorn seeds, isorhamnetin 3-*O*-β-glucoside-7-*O*-α-rhamnoside and serotonin (at concentrations of 1 and 50 μg/mL) on the exposition of GPIIb/IIIa on 10 µM ADP-stimulated platelets (**A**), GPIIb/IIIa on 20 µM ADP-stimulated platelets (**B**), P-selectin on 10 µM ADP-stimulated platelets (**C**), and P-selectin on 20 µM ADP-stimulated platelets (**D**) in human whole blood (*n* = 9–15; the blood for each repetition was collected from a different donor). Blood platelets were identified on the basis of CD61 exposition. 5000 CD61-positive objects were acquired for every sample. CD62P-PE antibody was used to assess the exposition of P-selectin on the surface of platelets. Results are expressed as the percentage of control. The numbers above the bars in graphs (**A**–**D**) represent the approximate % of inhibition. Data represent the means ± SD. The results were considered significant at *p* < 0.05 (* *p* < 0.05, ** *p* < 0.01). Figure (**E**) shows demonstrative diagrams of the exposition of GPIIb/IIIa on 20 µM ADP-stimulated blood platelets (for isorhamnetin 3-*O*-β-glucoside-7-*O*-α-rhamnoside—50 μg/mL). Figure (**F**) shows demonstrative diagrams of the exposition of P-selectin on 10 µM ADP-stimulated blood platelets (for isorhamnetin 3-*O*-β-glucoside-7-*O*-α-rhamnoside—50 μg/mL). The graphs on the left side show the gating of blood platelets based on the presence CD61-PerCP. The graphs on the right side show SSC/FITC diagrams (**E**), which demonstrate the percentage of PAC-1-FITC (PAC1 is an antigen present on the active form of GPIIb/IIIa) or SSC/PE diagrams (**F**), which demonstrate the percentage of CD62P-PE (P-selectin) positive objects per 5000 CD61 positive objects (blood platelets). Isorhamn.—isorhamnetin 3-*O*-β-glucoside-7-*O*-α-rhamnoside.

Figure 2 demonstrates the markers of blood platelet activation induced by 10 and 20 µM ADP, measured by flow cytometry. Isorhamnetin 3-*O*-β-glucoside-7-*O*-α-rhamnoside at two tested concentrations (1 and 50 µg/mL) induced changes in blood platelet activation, though they were not always statistically significant. Compared to the control, isorhamnetin 3-*O*-β-glucoside-7-*O*-α-rhamnoside at the concentration of 50 μg/mL inhibited the exposition of P-selectin on the surface of platelets stimulated by 10 μM ADP by about 24 ± 16% (*p* = 0.0055; Figure 2C). In blood platelets stimulated by 20 μM ADP, 1 and 50 μg/mL isorhamnetin 3-*O*-β-glucoside-7-*O*-α-rhamnoside inhibited the exposition of P-selectin by approximately 29 ± 24% (*p* = 0.0075) and 20 ± 19% (*p* = 0.0198), respectively (Figure 2D). It also decreased the levels of the active form of GPIIb/IIIa by approximately 33 ± 20% (at the concentration of 1 μg/mL; *p* = 0.0397) and 26 ± 13% (at the concentration of 50 μg/mL; *p* = 0.002) (Figure 2B). None of the tested fractions or compounds significantly affected the exposition of the active form of GPIIb/IIIa on the surface of platelets stimulated with 10 μM ADP (Figure 2A). Figure 2F shows flow cytometry diagrams of chosen samples (unstimulated blood platelets, platelets incubated with 10 μM ADP, and platelets incubated with 10 μM ADP and 50 μg/mL isorhamnetin 3-*O*-β-glucoside-7-*O*-α-rhamnoside). Figure 2E demonstrates flow cytometry diagrams of unstimulated blood platelets, platelets incubated with 20 μM ADP, and platelets incubated with 20 μM ADP and 50 μg/mL isorhamnetin 3-*O*-β-glucoside-7-*O*-α-rhamnoside. The diagrams depicted by Figure 2E,F show that stimulation with ADP increased the exposition of P-selectin and the active form of GPIIb/IIIa in comparison to unstimulated platelets; this effect was inhibited in the samples incubated with 50 μg/mL isorhamnetin 3-*O*-β-glucoside-7-*O*-α-rhamnoside.

### 3.2. Measurement of Thrombus Formation with T-TAS (PL-Chip) in Whole Blood

**Figure 3:**
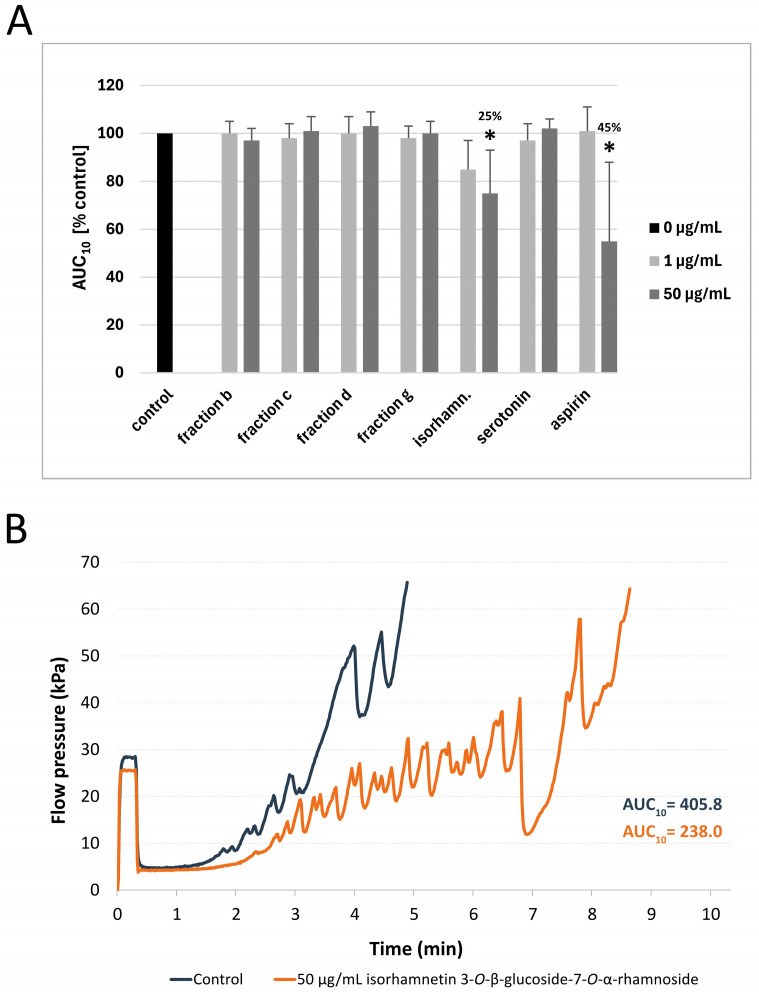
The effect of fraction b and fraction c from raw seeds, fraction d and fraction g from roasted sea buckthorn seeds, isorhamnetin 3-*O*-β-glucoside-7-*O*-α-rhamnoside and serotonin (at concentrations of 1 and 50 μg/mL) on thrombus formation in human whole blood (*n* = 6–11; the blood for each repetition was collected from a different donor). The measurements were carried out with T-TAS PL-chip (1500/s shear stress). The results are expressed as AUC_10_ (area under the curve). In the graph (**A**), AUC_10_ parameter is shown as a percentage relative to the control sample. The numbers above the bars in (**A**) represent the approximate % of inhibition. The data are expressed as means ± SD. The results were considered significant at *p* < 0.05 (* *p* < 0.05). Figure (**B**) demonstrates a selected diagram of the pressure recorded inside the PL-chip for 10 min (or until the occlusion time—60 kPa), for 50 μg/mL isorhamnetin 3-*O*-β-glucoside-7-*O*-α-rhamnoside). AUC—area under the curve; isorhamn—isorhamnetin 3-*O*-β-glucoside-7-*O*-α-rhamnoside.

The results of the T-TAS PL-chip test showed that none of the tested fractions significantly changed the AUC_10_ value in comparison to the control. However, isorhamnetin 3-*O*-β-glucoside-7-*O*-α-rhamnoside at the highest used concentration (50 µg/mL) demonstrated anti-platelet properties by significantly prolonging the occlusion time (*p* = 0.0173)—the AUC_10_ value was reduced by 25 ± 18%. In comparison, 50 μg/mL aspirin, which was used as a reference anti-platelet compound, decreased the AUC_10_ value by 45 ± 31% (*p* = 0.0494) (Figure 3A). Figure 3B shows a selected diagram of the pressure measured inside the PL-chip for 10 min. The pressure recorded in the sample incubated with 50 μg/mL isorhamnetin 3-*O*-β-glucoside-7-*O*-α-rhamnoside increased at a slower pace than in the control sample; it also took longer to reach the occlusion than in the control sample. The pressure diagrams of fractions b, c, d, and g, as well as serotonin (at the concentration of 50 μg/mL) can be found in Supplementary Figure S1.

### 3.3. Measurement of Blood Platelet Adhesion to Collagen and Fibrinogen

**Figure 4:**
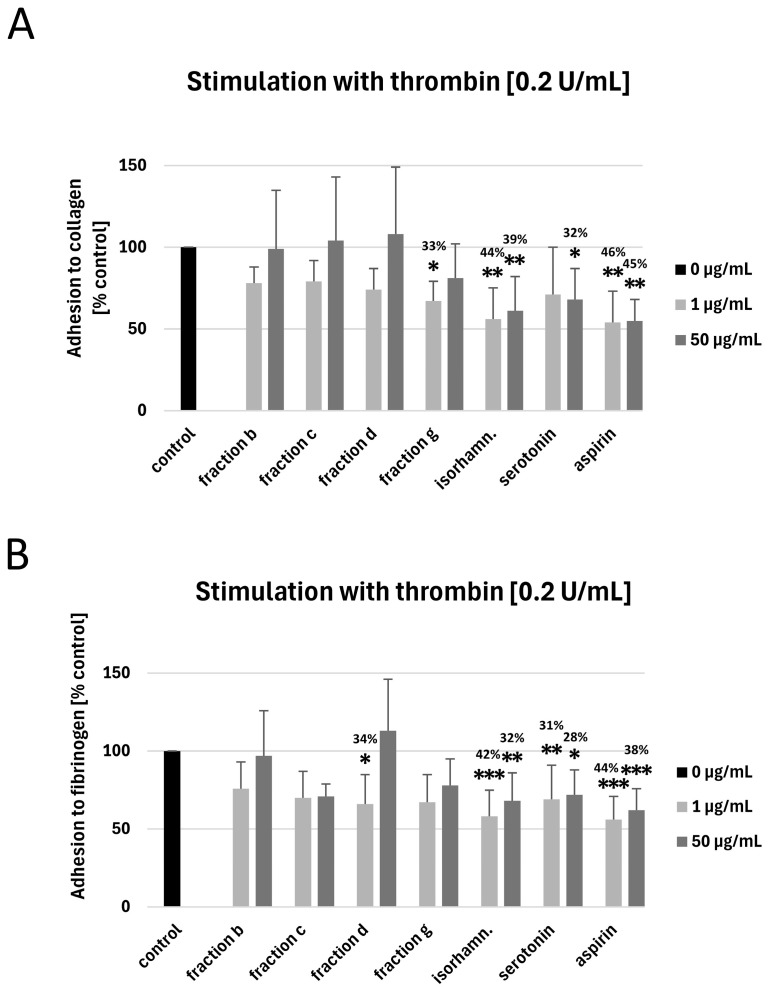
The effect of fraction b and fraction c from raw seeds, fraction d and fraction g from roasted sea buckthorn seeds, isorhamnetin 3-*O*-β-glucoside-7-*O*-α-rhamnoside and serotonin (at concentrations of 1 and 50 μg/mL) on the adhesion of thrombin-activated platelets to collagen (**A**) and fibrinogen (**B**) (*n* = 6–8; the blood for each repetition was collected from a different donor). Platelet adhesion is expressed as a percentage of the control sample. The numbers above the bars represent the approximated % of inhibition. Data represent the means ± SD. The results were considered significant at *p* < 0.05 (* *p* < 0.05, ** *p* < 0.01, *** *p* < 0.001). Isorhamn.—isorhamnetin 3-*O*-β-glucoside-7-*O*-α-rhamnoside.

None of the tested fractions at the highest used concentration (50 µg/mL) affected the adhesion of thrombin-activated platelets to collagen or fibrinogen (Figure 4A,B). However, fraction g at the concentration of 1 μg/mL decreased the adhesion of thrombin-activated blood platelets to collagen (by 33 ± 12%, *p* = 0.0476; Figure 4A), while fraction d at the concentration of 1 μg/mL inhibited the adhesion to fibrinogen by 34 ± 19% (*p* = 0.0436; Figure 4B). Moreover, isorhamnetin 3-*O*-β-glucoside-7-*O*-α-rhamnoside (at 1 and 50 μg/mL) inhibited the adhesion of thrombin-activated platelets to fibrinogen and collagen by 32–44%; the strength of activity was similar to that of aspirin (Figure 4A,B). Serotonin at both used concentrations (1 and 50 µg/mL) decreased the adhesion of thrombin-activated blood platelets to fibrinogen, while the adhesion to collagen was significantly inhibited only by 50 μg/mL serotonin (Figure 4A,B).

### 3.4. Measurement of COX-1 Activity

Fractions c and b (at the concentration of 50 μg/mL) inhibited the activity of COX-1 by approximately 87 and 61%, respectively. 50 μg/mL aspirin inhibited COX-1 by about 91%. On the other hand, isorhamnetin 3-*O*-β-glucoside-7-*O*-α-rhamnoside, fraction d, fraction g, and serotonin did not show inhibitory activity. The results of COX-1 inhibitor screening assay are shown in Table 1.

## 4. Discussion

Blood platelets play an important role in hemostasis, however, their hyperactivation can lead to aggregation at the site of atherosclerotic plaque rupture and thrombus formation, which promotes various CVDs. Blood platelet activation is induced by several physiological agonists, including thrombin, ADP, and collagen, among others. The first step of platelet activation is their adhesion to different adhesive proteins, including collagen. Stable platelet adhesion requires an interaction between collagen and its receptors: integrin α_2_β_1_ (GPIa/IIa), and glycoprotein (GP) VI. The binding of collagen to GPVI induces the release of agonists, such as thromboxane A_2_ (TXA_2_) and ADP, which leads to the activation of a major fibrinogen receptor—GPIIb/IIIa [34,35,36,37].

Hyperactivation of blood platelets is often reduced pharmacologically with anti-platelet drugs, such as COX-1 inhibitors (e.g., acetylsalicylic acid, also known as aspirin), inhibitors of P2Y_12_ receptors for ADP (e.g., clopidogrel), or intravenous GPIIb/IIIa inhibitors (e.g, abciximab). Unfortunately, prolonged use of these drugs is often associated with an increased risk of bleeding and other adverse effects. There is also a considerable group of patients that does not respond well to the often-used dual therapy with aspirin and clopidogrel and still suffers from thrombotic events [38,39,40,41]. Other potential molecular targets for new anti-platelet drugs include P2Y_2,_ thromboxane, and serotonin receptors, GPIb-IX-V-von Willebrand factor axis, P-selectin, CD40, glucagon-like peptide 1 (GLP-1), GPIa/IIa, GPIV or protease-activated receptor (PAR)-4 [41,42,43,44,45].

The literature data shows that some phenolic compounds (e.g., flavonoids) can exert cardioprotective activity and attenuate blood platelet activation by interacting with various platelet receptors [3]. One of the cardioprotective mechanisms of flavonoids involves their ability to neutralize reactive oxygen species (ROS), reducing oxidative stress, which plays a key role in the pathogenesis of cardiovascular diseases. They can also activate the nuclear factor erythroid 2-related factor 2 (Nrf2) pathway, enhancing the expression of antioxidant enzymes such as superoxide dismutase and catalase, which strengthens cellular defense against oxidative damage. Moreover, the presence of flavonoids in the diet is associated with lowered blood pressure, improved lipid profiles, and reduced risk of myocardial infarction [6]. Certain flavonoids demonstrate significant anti-platelet activity that could be utilized for cardiovascular disease prevention. One of their mechanisms of action involves the inhibition of cyclooxygenase enzymes, particularly COX-1, which leads to reduced production of important modulators of platelet function, such as pro-aggregatory thromboxane A_2_ [46,47]. A study conducted on human whole blood revealed that flavonoids with a catechol group in the B-ring (e.g., luteolin) effectively inhibit both COX-1 and COX-2, with the highest COX-1 selectivity observed in compounds containing fewer hydroxyl groups [47].

Isorhamnetin, a flavonoid found in various parts of *H. rhamnoides*, demonstrated cardioprotective properties [15,48]. It strongly reduced collagen-induced platelet aggregation in vitro (IC_50_ = 8.1 µM) and showed inhibitory activity when thrombin receptor activator peptide 6 (TRAP-6) and phorbol myristate acetate (PMA) were used as agonists (IC_50_ value was 16.1 µM for TRAP-6 and >100 µM for PMA) [49]. In another study, isorhamnetin suppressed platelet aggregation triggered by adenosine diphosphate or platelet-activating factor [50]. In in vitro and ex vivo rat models, isorhamnetin-containing extract (1.44 ± 0.46 mg/g) administered orally at 100 mg/kg reduced platelet aggregation by 33.2 ± 3.71%, which was comparable to aspirin (26.6 ± 4.45%). The authors suggest that the effective inhibition observed after oral administration indicates good bioavailability and metabolic stability of active components of the extract, including isorhamnetin. This indicates isorhamnetin could be good candidate for oral anti-platelet therapy [51]. Lei et al. (2024) [52] suggest that the anti-platelet activity of isorhamnetin might be mediated through the inhibition of mitochondrial bioenergetics. Moreover, isorhamnetin can modulate many molecular pathways including protein kinase B/phosphatidylinositol 3-kinase (AKT/PI3K), nuclear factor kappa B (NF-κB), or Ras/mitogen activated protein kinase (MAPK) [52].

Studies suggest that sea buckthorn fruits and seeds possess many health-promoting properties, including cardioprotective activity [17,53,54,55,56,57,58,59,60,61,62,63]. For example, Pang et al. (2008) found that total flavones from sea buckthorn seeds at a dose of 150 mg/kg/day have anti-hypertensive potential [17]. On the other hand, 4-week daily supplementation of sea buckthorn flavonol extract (0.4 g, containing 78 mg of flavonol aglycones) did not reduce CVDs risk markers in healthy people [62]. The anti-atherosclerosis effect of isorhamnetin (20 mg/kg/b.w.) isolated from sea buckthorn was observed in an in vivo model [64]. Other studies also indicated that isorhamnetin has anti-platelet properties [49,65,66].

Earlier in vitro results showed that the extracts from raw (no thermal processing) and roasted (thermally processed) sea buckthorn seeds have anti-platelet properties [24]. Therefore, the next course of action was to investigate two fractions (b and c) from raw sea buckthorn seed extract, and two fractions (d and g) from roasted sea buckthorn seed extract. For the first time, the results indicate that fraction g (isolated from roasted sea buckthorn seeds) has anti-adhesive potential—it inhibited the adhesion of thrombin-stimulated platelets to collagen. Fraction d, also derived from roasted sea buckthorn seeds reduced platelet adhesion to fibrinogen. In addition, the present study’s results indicate that serotonin, a dominant constituent of fraction c inhibited the adhesion of platelets stimulated by thrombin to fibrinogen. The results may suggest that the fractions from roasted seeds (d and g) are more effective anti-adhesive factors than fractions from raw sea buckthorn seeds. Moreover, this study provides novel evidence that isorhamnetin 3-*O*-β-glucoside-7-*O*-α-rhamnoside isolated from sea buckthorn seeds possesses anti-adhesive potential in washed blood platelets.

Both flow cytometry and T-TAS were used to investigate platelet activation in whole blood, which is a more natural environment than media used for maintaining platelets after isolation. The T-TAS PL chip assesses primary hemostasis and can be used to monitor the efficiency of individual and combined anti-platelet drugs or supplements in patients with cardiovascular diseases [30,67]. However, a limitation of this method is that it does not provide information about individual blood platelet activation pathways. For this reason, other methods should be employed alongside T-TAS.

Neither of the tested fractions at any of the tested concentrations (1 and 50 µg/mL) significantly impacted the exposition of P-selectin and the active form of GPIIb/IIIa on blood platelets activated by ADP (10 and 20 µM). However, the results may indicate that a pure chemical compound isolated from sea buckthorn seeds—isorhamnetin 3-*O*-β-glucoside-7-*O*-α-rhamnoside—is a more effective anti-platelet factor in whole blood than the tested fractions, as it could inhibit the exposition of both P-selectin and GPIIb/IIIa. It also significantly increased the time needed for thrombus formation, which was measured by the T-TAS PL-chip. None of the tested fractions had any effect on thrombus formation; this indicates that the content of isorhamnetin 3-*O*-β-glucoside-7-*O*-α-rhamnoside in fraction b was insufficient to provide an observable effect.

The results of the COX-1 assay indicate that isorhamnetin 3-*O*-β-glucoside-7-*O*-α-rhamnoside is not a COX-1 inhibitor, suggesting that its anti-platelet activity is mediated by other molecular mechanisms. However, both fractions isolated from raw seeds (b and c) showed inhibitory activity. Even though isorhamnetin 3-*O*-β-glucoside-7-*O*-α-rhamnoside and serotonin were among the major components of fractions b and c, respectively, they do not necessarily have to be responsible for their inhibitory activity; the fractions contain many other active compounds, which, though less prominent, can act together to produce a stronger effect.

Apart from isorhamnetin derivatives, fraction b also contains various glycosides of kaempferol and quercetin. Zaragozá et al. (2022) showed that quercetin is a strong cyclooxygenase-1 inhibitor—at a concentration of 1.5 mM it decreased the activity of COX-1 by approximately 90% [68]. In another study, kaempferol and its glycosides inhibited COX-1 activity as well [69]. This suggests that these compounds might be responsible for the activity of fraction b. Apart from serotonin, which did not inhibit COX-1, fraction c contains diverse B-type procyanidins. An in silico study suggested that procyanidins from tamarind can inhibit COX-1, but more research is needed to ascertain if this is indeed the case [70].

Serotonin is an ancient indoleamine with important functions in both animals and plants. It is not only a neurotransmitter but also a peripheral hormone transported by blood platelets, though it cannot cross the blood–brain barrier [71,72]. Platelets store 5-HT in dense granules, releasing it after activation. It causes vasoconstriction and amplifies platelet activation at the site of blood vessel injury. Serotonin binds to membrane 5-HT receptors (5-HT2AR) on the surface of platelets, amplifying their functional responses (e.g., aggregation) [72,73]. 5-HT2A receptor activation also initiates the uptake of serotonin into platelets by serotonin transporters (SERT); this is an important mechanism of regulating plasma 5-HT levels, which are maintained at a low nanomolar range [73,74]. 5-HT itself is a weak platelet agonist, but it can amplify the signaling induced by other agonists, such as collagen, ADP, and epinephrine [72]. On the other hand, a study by Duerschmied et al. (2009) [73] showed that serotonin can decrease platelet adhesion to collagen-bound von Willebrand factor under arterial shear conditions (1500 s^−1^). 5-HT initiated the shedding of adhesion receptor glycoprotein GPIbα, which was mediated by 5-HT2AR stimulation and subsequent activation of tumor necrosis factor-alpha-converting enzyme (TACE) [73]. In a study by Takahashi et al. [75], serotonin did not inhibit COX-1 (IC_50_ > 400 μM), which is consistent with the results of the COX-1 screening assay presented here [75].

The anti-adhesive activity of fraction d may be due to its content of triterpenoid saponins. Other authors also reported their anti-platelet activity. For example, Zuo et al. [76] noted that ginsenosides Rb2 (G-Rb2), and Rd2 (G-Rd2)—triterpenoid saponins derived from *Panax notoginseng* root—significantly reduced human blood platelet activation induced by ADP in vitro. The 50% inhibitory concentration (IC_50_) value of G-Rb2 and G-Rd2 against blood platelet aggregation induced with ADP was 85.5 ± 4.5 μg/mL and 51.4 ± 4.6 μg/mL, respectively. Authors suggest that G-Rd2 and G-Rb2 could modulate platelet P2Y_12_-mediated signaling by upregulating cAMP/PKA and downregulating PI3K/Akt/Erk1/2 signaling pathways [76]. Moreover, the results of Irfan et al. [77] indicate that G-Rp3 modulates agonist-induced blood platelet activation by inhibiting granule secretion, GPIIb/IIIa activation, MAPK signaling, Src, PI3K/Akt, and PLCγ2 activation, and VASP stimulation [77]. Ouyang et al. (2019) also found that five 11α, 12α-epoxy pentacyclic triterpenoid saponins from *Glechoma longituba* have anti-platelet activities, including anti-aggregatory potential [78]. Other triterpenoid saponin—platycodin D also inhibited blood platelet function and thrombus formation through the internalization of platelet glycoprotein receptors [79]. On the other hand, Zhang et al. [80] found that 20(S)-panaxdiol is an important hemostatic agent in *Panax notoginseng*. It could induce platelet aggregation by modulating PI3K/Akt/GSK3β pathway activation and calcium signaling [80].

**Figure 5:**
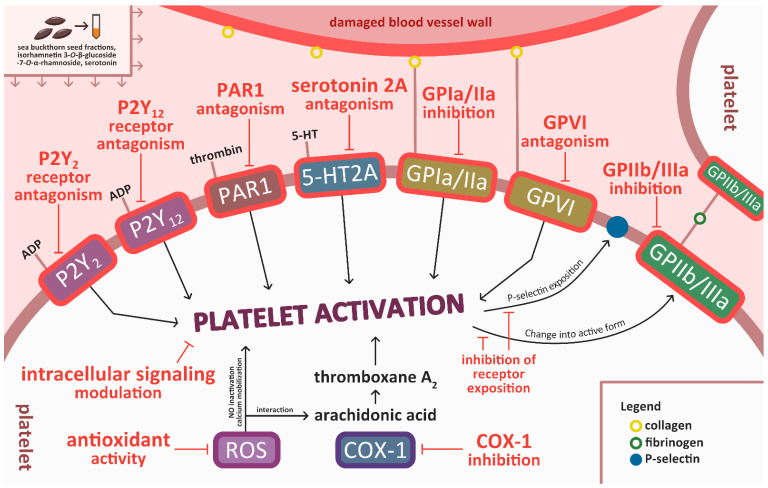
Potential mechanisms of action of isorhamnetin 3-*O*-β-glucoside-7-*O*-α-rhamnoside, serotonin, and other compounds from sea buckthorn seed fractions. 5HT2A—serotonin receptor 2A; ADP—adenosine diphosphate; COX-1—cyclooxygenase-1; GP—glycoprotein; NO—nitric oxide; PAR1—protease-activated receptor 1; ROS—reactive oxygen species. Compilation of data from [41,42,43,44,82].

Earlier results indicate that isorhamnetin derivatives inhibited adhesion of not only blood platelets stimulated by thrombin, but also unstimulated platelets [65]. This suggests that these phenolic compounds might interact with thrombin receptors (for example protease-activated receptor 1 (PAR-1) or PAR-4) and inhibit their activities, but more research is needed to confirm this. PAR-1 and PAR-4 mediate most of the platelet response to thrombin; PAR-1 is responsible for platelet activation at low thrombin concentrations, while PAR-4 acts at higher thrombin concentrations [35]. Thrombin causes an increase in cytosolic Ca^2+^ levels, which triggers Ca^2+^-dependent signaling, including phospholipase A_2_ activation [81]. Apart from thrombin receptor inhibition, it is possible that compounds present in sea buckthorn seeds exert their anti-platelet activity through other mechanisms. For example, isorhamnetin 3-*O*-β-glucoside-7-*O*-α-rhamnoside inhibited P-selectin and GPIIb/IIIa exposition in platelets stimulated with ADP. This action could perhaps be achieved through P2Y_2_ and P2Y_12_ (receptors for ADP) inhibition, or by affecting cellular signaling pathways within the cell [41]. Other surface receptors, for example GPIa/IIa (α_2_β_1_) or GPVI, which play an important role in platelet adhesion and activation by binding to collagen [43] could also be targeted by compounds from sea buckthorn seeds. GPIa/IIa and GPIV bind collagen directly, while GP IIb/IIIa and GP Ibα can bind to collagen through vWF. GPIV is the most potent signaling receptor among them [81]. In a previous study, fractions from both raw and roasted sea buckthorn seeds and serotonin showed antioxidant activity [25]. Antioxidants can show anti-platelet effects by inhibiting ROS production. ROS are crucial in platelet activation—they act as second messengers through nitric oxide (NO) inactivation or interaction with arachidonic acid [82]. Moreover, the decrease in ROS levels can lead to the reactivation of phosphatases, which inhibit the activity of tyrosine kinases, preventing the exposition of GPIIb/IIIa on the platelet surface [83]. Potential mechanisms of action of isorhamnetin 3-*O*-β-glucoside-7-*O*-α-rhamnoside, serotonin, and other compounds from sea buckthorn seed fractions are depicted in Figure 5.

The concentrations used in this study (1 and 50 μg/mL) can generally be achieved in blood through oral supplementation of phenolic compounds [84,85,86]. However, despite the promising in vitro anti-platelet properties of isorhamnetin 3-*O*-β-glucoside-7-*O*-α-rhamnoside, poor bioavailability of various phenolic compounds might be an obstacle in achieving the same results in vivo. Moreover, the response of individual patients to the same drug can be different due to the variations in their metabolism. Isorhamnetin has poor water solubility, which limits its absorption in the gastrointestinal tract; however, its bioavailability can be enhanced by various formulations, including phytic acid or phospholipid complexes [87,88,89]. A pharmacokinetic study conducted on rats determined that after intravenous injection of 0.5 mg/kg isorhamnetin, its half-life of the elimination phase was approximately 1 h; the maximum concentration in plasma was 1431 ng/mL. Moreover, in vitro incubation (37 °C, 60 min) of 30 μM isorhamnetin solution (dissolved in 1% *v*/*v* DMSO) with platelet-rich plasma showed that less than 1% of this compound penetrated platelets [90]. Intragastric administration of isorhamnetin at a dose of 50 mg/kg resulted in the maximum concentration of 0.346 mg/L in rat plasma. The elimination half-life was 6.7 h [91]. The bioavailability of various triterpenoid saponins is also relatively low, but it can sometimes be improved by microbial biotransformation [92]. Therefore, isorhamnetin 3-*O*-β-glucoside-7-*O*-α-rhamnoside, triterpenoid saponins, and other bioactive compounds from sea buckthorn seeds need to be evaluated in vivo as well. For now, the lack of in vivo data limits the conclusions that can be drawn from this study. It is essential to analyze the concentration of circulating phenolic compounds and their metabolites and assess whether the concentrations used in this study correspond to physiologically achievable levels.

Future in vivo studies are required to clarify the anti-platelet mechanism of action at physiologically relevant concentrations and confirm the therapeutic potential of sea buckthorn seeds and their bioactive compounds in cardiovascular diseases. It is also worthwhile to assess the synergistic or antagonistic effects of serotonin and isorhamnetin 3-*O*-β-glucoside-7-*O*-α-rhamnoside with other compounds within the fractions. A thorough understanding of circulating concentrations, cellular targets, and mechanisms of action is essential in advancing the development of anti-platelet supplements from sea buckthorn seeds; these subjects are recommended for future studies involving sea buckthorn seeds or isorhamnetin 3-*O*-β-glucoside-7-*O*-α-rhamnoside. Importantly, sea buckthorn seeds could be used as an addition to bread and cake without losing their anti-platelet properties. This knowledge will contribute to the development of preventive and therapeutic strategies against CVDs.

## 5. Conclusions

Isorhamnetin 3-*O*-β-glucoside-7-*O*-α-rhamnoside, a flavonoid present in sea buckthorn seeds shows promising in vitro anti-platelet activity, which makes it a potential candidate for CVD prevention, but more data about its bioavailability, in vivo efficiency, safety, and mechanisms of action is needed to decide whether it would be useful as an anti-platelet agent. Fractions d and g isolated from roasted sea buckthorn seeds, and serotonin exhibited antiadhesive potential in vitro but did not modulate platelet activation or thrombus formation; this indicates that isorhamnetin 3-*O*-β-glucoside-7-*O*-α-rhamnoside is a more effective anti-platelet agent in full blood than sea buckthorn seed fractions.

## Figures and Tables

**Table 1:** Inhibition of COX-1 activity by tested fractions and tested compounds. % of inhibition is a mean from two dilutions, each measured in duplicate. The fractions and tested compounds were incubated with COX-1 for 30 min, at the concentrations of 1 and 50 μg/mL.

Concentration	Inhibition of COX-1 [%]						
	Fraction				Isorhamnetin 3-*O*-β-glucoside-7-*O*-α-rhamnoside	Serotonin	Aspirin
	b	c	d	g			
1 μg/mL	31	0	−49	−23	−31	−6	−22
50 μg/mL	61	87	−10	−45	−1	−66	91

## Data Availability

The datasets generated during and/or analysed during the current study are available from the corresponding author on reasonable request.

## Supplementary Materials

The following supporting information can be downloaded at: https://www.mdpi.com/article/10.3390/nu17193074/s1, Figure S1: Diagrams of the pressure recorded inside the PL-chip for 10 min (or until the occlusion time—60 kPa), for 50 μg/mL fraction b, c, d, g, and serotonin.

## Abbreviations

5-HT—5-hydroxytryptamine, serotonin; 5-HT2AR—5-HT 2A receptor; ADP—adenosine diphosphate; AUC—area under the curve; BAPA—benzyl-sulfonyl-D-Arg-Pro-4-amidinobenzylamide; BSA—bovine serum albumin; COX-1—cyclooxygenase 1; CPDA—citrate/phosphate/dextrose/adenine; CVDs—cardiovascular diseases; GLP1—glucagon-like peptide 1; GP—glycoprotein; G-Rb2—ginsenosides Rb2; G-Rd2—ginsenosides Rd2; Nrf2—nuclear factor erythroid 2-related factor 2; PAR1—protease-activated receptor 1; PBS—phosphate-buffered saline; PMA—phorbol myristate acetate; ROS—reactive oxygen species; SERT—serotonin transporters; TACE—tumor necrosis factor-alpha-converting enzyme; TRAP-6—thrombin receptor activator peptide 6; T-TAS—Total Thrombus-formation Analysis.
