# Supplementary material for: Fractions from Sea Buckthorn Seeds and Their Bioactive Ingredients as Modulators of Human Blood Platelet Response In Vitro: The Role of Thermal Processing
Source: Nutrients. 2025 Sep 27;17(19):3074. doi: 10.3390/nu17193074 (PMC12526294; doi:10.3390/nu17193074)
Supplement: Supplementary file 1 [file nutrients-17-03074-s001.zip › nutrients-3875222-supplementary.pdf]

S1

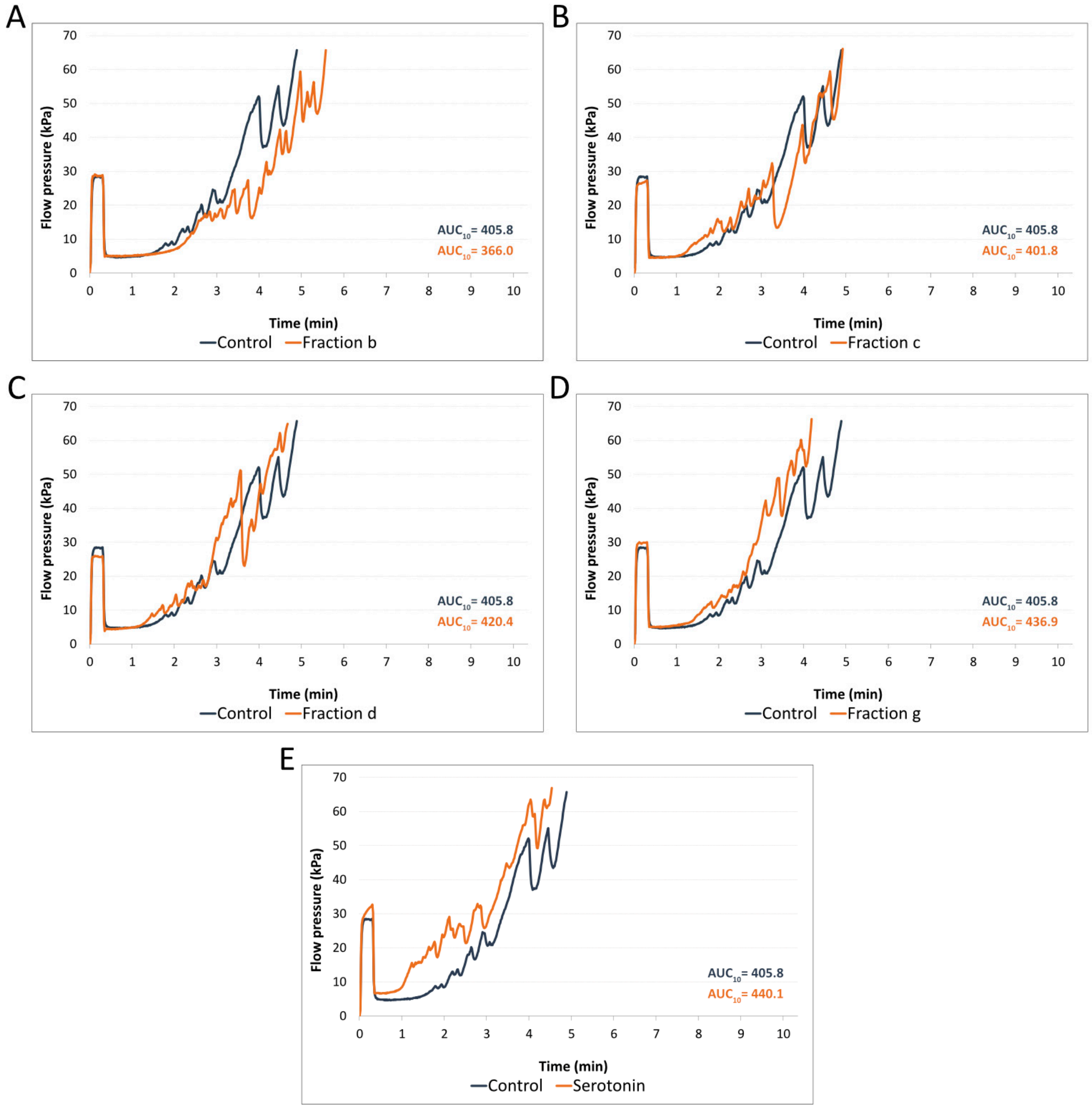

Supplementary Figure S1 (S1). Diagrams of the pressure recorded inside the PL-chip for 10 minutes (or until the occlusion time - 60 kPa), for 50 μg/mL fraction b, c, d, g, and serotonin.
